# Knockdown of lnc-KCNC3-3:1 Alleviates the Development of Atherosclerosis *via* Downregulation of JAK1/STAT3 Signaling Pathway

**DOI:** 10.3389/fcvm.2021.701058

**Published:** 2021-09-03

**Authors:** Limin Sun, Xin He, Tao Zhang, Guizhou Tao, Xin Wang

**Affiliations:** ^1^Department of General Practice, The First Affiliated Hospital of Jinzhou Medical University, Jinzhou, China; ^2^Department of Cardiology, The First Affiliated Hospital of Jinzhou Medical University, Jinzhou, China

**Keywords:** atherosclerosis, lnc-KCNC3-3:1, apoptosis, triglyceride, JAK1/STAT3

## Abstract

**Background:** Atherosclerosis is a major cause of coronary artery disease (CAD), and CAD is one of the main causes leading to death in most countries. It has been reported that lncRNAs play important roles in the development of atherosclerosis; thus, we aimed to explore lncRNAs that are closely related to the occurrence and development of atherosclerosis.

**Methods:** The data GSE113079 from the GEO database was used to explore the dysregulated lncRNAs in peripheral blood mononuclear cells (PBMCs) between 93 patients with CAD and 48 healthy controls. Next, RT-qPCR was performed to detect the level of lncRNAs in HUVEC cells and CCK-8 was performed to detect cell viability. Then, flow cytometry assays were used to determine the apoptosis of HUVEC. In addition, ELISA assay was used to measure the concentrations of triglyceride (TG), low density lipoprotein cholesterin (LDL-C), and high density lipoprotein cholesterol (HDL-C). Moreover, western blot assay was used to detect the expression of proteins.

**Results:** lnc-KCNC3-3:1 was significantly upregulated in PBMCs of patients with CAD. In addition, oxidized low density lipoprotein (oxLDL) notably inhibited the proliferation and induced the apoptosis of HUVEC, while this phenomenon was notably reversed by lnc-KCNC3-3:1 knockdown. Moreover, oxLDL significantly promoted the migration of HUVECs, which was significantly restored by knockdown of lnc-KCNC3-3:1. Moreover, lnc-KCNC3-3:1 siRNA1 could reverse oxLDL-induced HUVEC growth inhibition, and lnc-KCNC3-3:1 silencing could inhibit the expressions of p-JAK1 and p-STAT3 in oxLDL-treated HUVECs. Animal study revealed that knockdown of lnc-KCNC3-3:1 alleviated the symptom of atherosclerosis, and it could inhibit the expressions of p-JAK1, p-STAT3 and p-Akt in tissues of atherosclerosis mice.

**Conclusion:** Knockdown of lnc-KCNC3-3:1 alleviates the development of atherosclerosis via downregulation of JAK1/STAT3 signaling pathway. These data indicated that lnc-KCNC3-3:1 might serve as a potential target for the treatment of atherosclerosis.

## Introduction

Atherosclerosis (AS) is the main cause of the development of cardiovascular and cerebrovascular diseases (1–3). It is characterized by early local damage to the intima of the artery, lipid deposition, and then intima fibrous tissue hyperplasia, local intima thickening, and the formation of plaque (1–3). Atherosclerosis can be caused by a number of factors, including dyslipidemia, high blood pressure, smoking, diabetes and impaired glucose tolerance, genetic factors, and so on (4, 5). Human umbilical vein endothelial cells (HUVECs) regulate the vascular tension and participate in lipoprotein metabolism (6). Evidence has shown that HUVEC damage is an important feature of early atherosclerotic lesions as dysfunctions of HUVECs including inappropriate proliferation, migration and apoptosis are critical cellular events resulting to the occurrence and development of atherosclerosis (1). Therefore, maintaining the function of HUVECs is crucial for the treatment of atherosclerosis.

Long non-coding RNA (LncRNA, lncRNA) is a class of RNA molecules with a transcript length of >200 nt (7, 8). It is generally believed that they do not encode proteins, but participate in the regulation of protein-coding genes at various levels (epigenetic regulation, transcriptional regulation and post-transcriptional regulation, etc.) (7). Meanwhile, lncRNA plays an important regulatory role in the occurrence and development of atherosclerosis (1). For example, Pan et al. found that the expression of lncRNA H19 is significantly upregulated in the patients with atherosclerosis (1). Li et al. indicated that lncRNA TUG1 could promote the progression of atherosclerosis *via* regulating miRNA-21/PTEN pathway (9). However, more lncRNAs involved in progression of atherosclerosis are needed to be explored. In the current study, we investigated the dysregulated lncRNAs in peripheral blood mononuclear cells (PBMCs) between 93 patients with CAD and 48 healthy controls. We hope the findings may provide some theoretical basis for the diagnosis and treatment of atherosclerosis.

## Materials and Methods

### Bioinformatics Analysis

Atherosclerosis-related datasets (GSE113079) were downloaded from the Gene Expression Omnibus (GEO, https://www.ncbi.nlm.nih.gov/geo/) database. R language was used to analyze the differentially expressed genes (DEGs) in PBMCs of 93 patients with CAD and 48 healthy controls. *P* < 0.05 and |log2 (FC)| > 2 were set as the threshold. LncRNA-related genes were analyzed by Gene Set Enrichment Analysis (GSVA) method to obtain their related pathways.

### Cell Culture

Human umbilical vein endothelial cell (HUVEC) was obtained from American Type Culture Collection (ATCC, Rockville, MD, USA). HUVEC cells were maintained in DMEM medium containing 10% FBS, penicillin 100 U/ml, and streptomycin 100 mg/ml in a humidified incubator containing 5% CO_2_ at 37°C.

### Reverse Transcription-Quantitative Polymerase Chain Reaction (RT-qPCR)

Trizol reagent (ELK Biotechnology, Wuhan, China) was used to extract the RNA from cells. RNA was reversed transcribed into cDNA using an EntiLink™ 1st Strand cDNA Synthesis Kit (ELK Biotechnology). Next, RT-qPCR was performed using the EnTurbo™ SYBR Green PCR SuperMix (ELK Biotechnology, EQ001) on the StepOne™ Real-Time PCR (Life technologies, Carlsbad, California, USA). The primers were as follows: H-ACTIN, forward, 5′-GTCCACCGCAAATGCTTCTA-3′, reverse, 5′-TGCTGTCACCTTCACCGTTC-3′; H-LOC100129516, forward, 5′-GCTGTGGTTATCCAATCCCTC-3′, reverse, 5′-GGCAAATGACTTCACCTCCC-3′; H-lnc-KCNC3-3:1, forward, 5′-GAAGATTGGGAACCGACAACA-3′, reverse, 5′-TTGCATCGAGAATCGTGCTC-3′; ENSG00000236780.1, forward, 5′-GATGCTCTCCACACCAGTCCA-3′, reverse, 5′-TGTGAACAGGGCTGGAATGAG-3′; ENSG00000205959.3, forward, 5′-GCTGTCCGAGCAAATGTCTCT-3′, reverse, 5′-GTGGCACTGCTGAGAAGAGGA-3′; ENSG00000261482.1, forward, 5′-GGGCTGCTGACTTCCACAG-3′, reverse, 5′-AATATCCACTCTGGGTGCAGC-3′. β-actin acted as the inner control. The 2^−ΔΔCT^ method was used for data analysis.

### Flow Cytometry Assay

Annexin-V-FITC apoptosis detection kit (AO2001-02P-G, Tianjin Sanjian Biotechnology Co., Ltd., Tianjin, China) was used to detect cell apoptosis. Briefly, HUVECs were maintained in DMEM medium and cultured in six-well plate (51 × 0^4^/ml). After treatments, the cells were stained with 5 μl Annexin-V-FITC and 5 μl PI staining solution for 15 min in darkness. Finally, a flow cytometer was used to analyze the cell apoptosis.

### Cell Transfection

SiRNA against lncRNAs (LOC100129516 siRNA1, LOC100129516 siRNA2, LOC100129516 siRNA3; lnc-KCNC3-3:1 siRNA1, lnc-KCNC3-3:1 siRNA2, lnc-KCNC3-3:1 siRNA3; ENSG00000261482.1 siRNA1, ENSG00000261482.1 siRNA2, ENSG00000261482.1 siRNA3; siRNA-ctrl) were purchased from RiboBio (Guangzhou, China).

LOC100129516 siRNA1, LOC100129516 siRNA2, LOC100129516 siRNA3; lnc-KCNC3-3:1 siRNA1, lnc-KCNC3-3:1 siRNA2, lnc-KCNC3-3:1 siRNA3; ENSG00000261482.1 siRNA1, ENSG00000261482.1 siRNA2, ENSG00000261482.1 siRNA3 or siRNA-ctrl was transfected into HUVEC cells using Lipofectamine® 2000 (Thermo Fisher Scientific, Waltham, MA, USA).

The sequences of siRNAs were presented as following: LOC100129516 siRNA1, 5′-CCCAGGCTACCATCCCTCCAAATAA-3′; LOC100129516 siRNA2, 5′-CACCATTCACTTTCCGGCAGCTTAG-3′; LOC100129516 siRNA3, 5′-CCGCTCCTTCTTGTCAGAGTAAGTA-3′; siRNA-ctrl, 5′-CCCATCGTACCTCCCAACCAGATAA-3′; lnc-KCNC3-3:1 siRNA1, 5′-ACAACATGCGTGGGAGGCGACAGGT-3′; lnc-KCNC3-3:1 siRNA2, 5′-ACAGGTCGCGACGGCAGCCACAGGT-3′; lnc-KCNC3-3:1 siRNA3, 5′-GAGGCTCCCTGAACCACCCTCCCTC-3′; siRNA-ctrl, 5′-ACACGTAGGTGGGAGCAGCACAGGT-3′; ENSG00000261482.1 siRNA1, 5′-TGGCTGCTGGAGAGGATTCTCACAA-3′; ENSG00000261482.1 siRNA2, 5′-GGCTGCTGGAGAGGATTCTCACAAA-3′; ENSG00000261482.1 siRNA3, 5′-GCTGCTGGAGAGGATTCTCACAAAG-3′; siRNA-ctrl, 5′-GCTGCTGTCAAGGATTCTGGAGCAA-3′.

### Cell Viability Assay

HUVEC cell viability was determined by Cell Counting Kit-8 (CCK8, Beyotime, Shanghai, China). The cells were placed into 96-well plate at density of 51 × 0^3^/ well. After treatments, the cells were incubated with 10 μL CCK-8 solution at 37C for 4 h. Then, the absorbance at 450 nm was detected using a spectrophotometer.

### Specimen Collection

The plasma samples were collected from five patients with CAD and five healthy donators in the First Affiliated Hospital of Jinzhou Medical University. Plasma was stored in liquid nitrogen immediately after collection. This study was confirmed by the Ethics Committee of the First Affiliated Hospital of Jinzhou Medical University, and each patient's written informed consent was obtained. Meanwhile, the information of healthy donators and patients with CAD were presented in Table 1. All these five CAD patients received coronary artery bypass grafting.

**Table 1:** The information of clinical sample donators.

**Participant**	**Age**	**Gender**
Health control 1	58	Male
Health control 2	54	Female
Health control 3	56	Male
Health control 4	64	Male
Health control 5	62	Male
CAD patient 1	54	Male
CAD patient 2	62	Male
CAD patient 3	64	Female
CAD patient 4	57	Male
CAD patient 5	57	Male

### Western Blot Assay

Protein Lysis Buffer (Beyotime, Shanghai, China) was used to extract the protein from cells or arterial tissues of mice. BCA kit (Nanjing Jiancheng Biogngineering Institute Nanjing, China) was used to detect the protein concentration. Then, 30 μg proteins were separated by 10% sodium dodecyl sulfate-polyacrylamide gel electrophoresis (SDS-PAGE) and transferred to polyethylenedi fluoride (PVDF). The membrane was blocked with 3% skim milk for 1 h at room temperature followed by the incubation of the primary antibodies at 4°C overnight. The primary antibodies used as follows: anti-Bax (1:1000, Abcam, Cambridge, MA, USA), anti-p-Akt 1:1000, Abcam), anti-Bcl-2 (1:1000, Abcam), anti-Cleaved caspase 3 (1:1000, Abcam), anti-p-JAK1 (1:1000, Abcam), anti-JAK1 (1:1000, Abcam), anti-p-STAT3 (1:1000, Abcam), anti-STAT3 (1:1000, Abcam), anti-β-actin (1:1000, Abcam). The β-actin worked as an internal control. Next, the membranes were incubated with goat anti-rabbit or mouse IgG secondary antibodies (1:5000, Abcam) for 1 h at room temperature. Finally, the protein bands were detected using an efficient chemiluminescence (ECL) kit (Thermo Fisher Scientific).

### Transwell Assay

Transwell chambers (Corning, New York, NY, USA) with a pore size of 8 μm were used to detect the migration ability of HUVEC. The Cells (11 × 0^5^) were added into the upper chamber suspended in 200 μL serum-starved medium. Next, the lower chamber was added with 600 μL of DMEM medium containing 10% FBS. After 24 h of incubation, the cells on the lower membrane surface were stained with 1% crystal violet for 30 min and pictured with a fluorescence microscope.

### *In vivo* Model of Atherosclerosis

Male ApoE^−/−^ mice (aged 7 weeks) were purchased from Vital River (Beijing, China) and housed in a specific-pathogen-free animal facility. To establish atherosclerosis model *in vivo*, these mice were fed a high-fat diet containing 60% of total calories from fat [D12492; HFK Bioscience (Beijing, China)] or a standard 4% fat diet (D12450B; HFK Biological Sciences) for 12 weeks. An lnc-KCNC3-3:1 knockdown mouse model was constructed by injecting a lentivirus (Lenti) expressing RNA inference (RNAi) targeting lnc-KCNC3-3:1 (lnc-KCNC3-3:1 Lenti-siRNA1), which was purchased from Genepharma (Shanghai, China). Next, lnc-KCNC3-3:1 Lenti-siRNA1 was injected into the tail vein of mice in atherosclerosis + lnc-KCNC3-3:1 siRNA1 group. Finally, mice were sacrificed for collection of arterial tissues and serum. All animal procedures were approved by the Committee of the First Affiliated Hospital of Jinzhou Medical University. The National Institute of Health Guide for the Care and Use of Laboratory Animals was strictly followed (10).

### Oil-Red O and H&E Staining of Artery

Arteries of mice were perfused with PBS, and the arteries were carefully separated. Then, the mouse arteries were stained with Oil-red O and H&E staining, according to previous literature (11).

### ELISA Assay

The levels of triglyceride (TG), low-density lipoprotein cholesterol (LDL-C) and high-density lipoprotein cholesterol (HDL-C) in the plasma of mice were measured with ELISA assays, respectively. These ELISA assays were obtained from Nanjing Jiancheng Bioengineering Institute (Nanjing, China).

### Statistical Analysis

All experiments were repeated three times. Statistical analyses were performed using GraphPad Prism software (version 7.0, La Jolla, CA, USA). The measurement data were shown as the mean ± SD. The one-way analysis of variance was used for comparison between multiple groups and Tukey's test was used for comparison between the two groups. *P* < 0.05 was regarded as statistically significant differences.

## Results

### Identification of DEGs Between Patients With CAD and Healthy Controls

**Figure 1:**
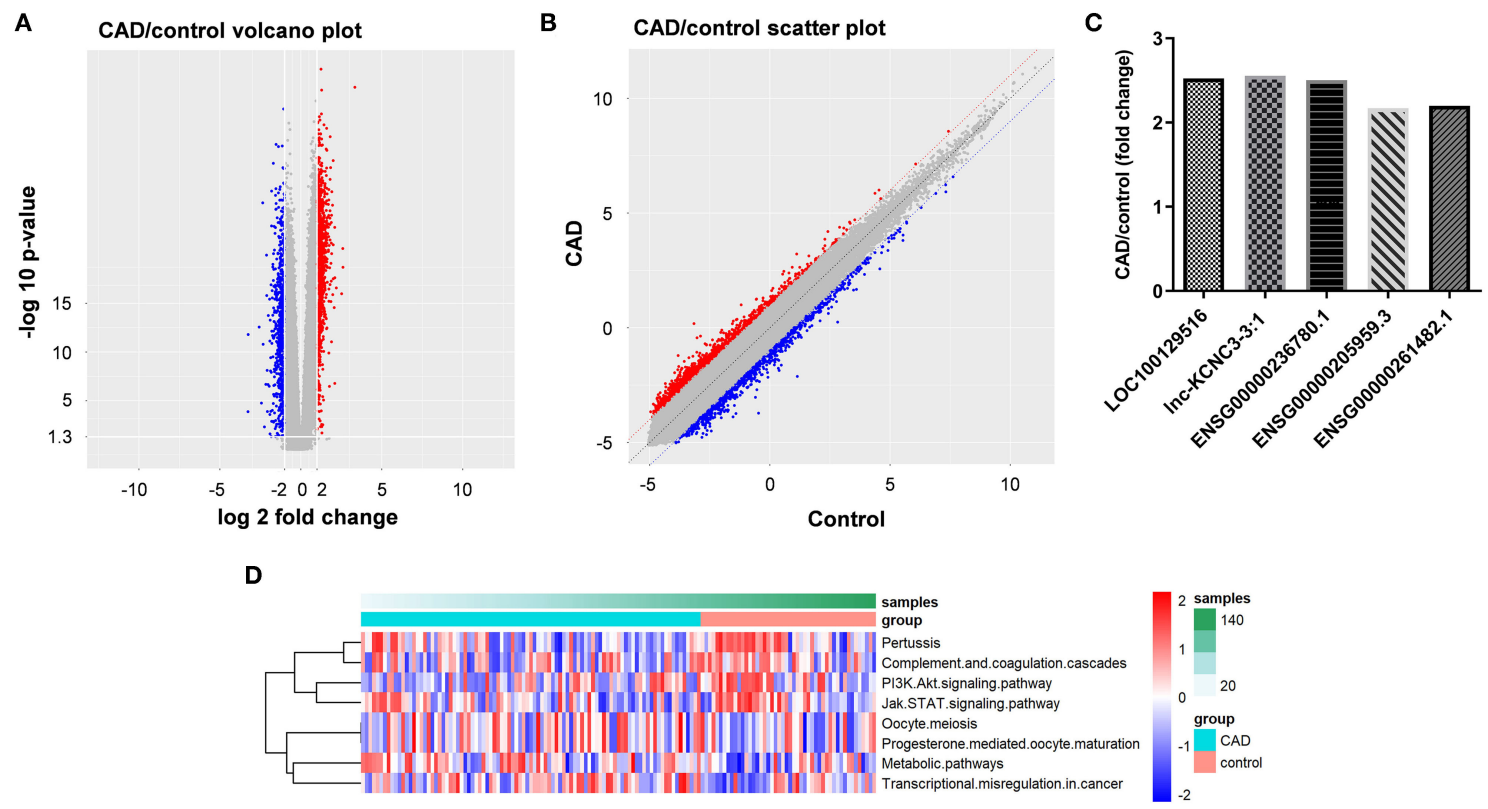
Identification of DEGs between patients with CAD and healthy controls. **(A,B)** DEGs between patients with CAD and healthy controls were analyzed by R language. The DEGs were presented in volcano plot and scatter plot. The red dots represent genes that are significantly upregulated, and the blue dots represent genes that are significantly downregulated. **(C)** LncRNA-related genes were analyzed by GSVA method to obtain their related pathways. **(D)** RT-qPCR was used to detect the expression levels of LOC100129516, lnc-KCNC3-3:1, ENSG00000236780.1, ENSG00000205959.3 and ENSG00000261482.1.

To explore the lncRNAs that are closely related to the occurrence and development of atherosclerosis, we analyzed the dataset GSE113079. As shown in Figures 1A,B, volcano plot and scatter plot revealed the significantly upregulated (red dots) or downregulated (blue dots) lncRNAs. The top five differentially expressed lncRNA were presented in Figure 1C. The co-expression relationship between differentially expressed lncRNA and mRNA genes was calculated in all samples, and the co-expression of mRNA and lncRNA was obtained according to *P* < 5E−63. Next, the differentially expressed lncRNA-related pathways were explored by gene set variation analysis (GSVA) (12). As indicated in Figure 1D, the differentially expressed lncRNAs were mainly involved in PI3K and JAK signaling pathways.

### OxLDL Increases the Expression of LOC100129516, lnc-KCNC3-3:1 and ENSG00000261482.1

**Figure 2:**
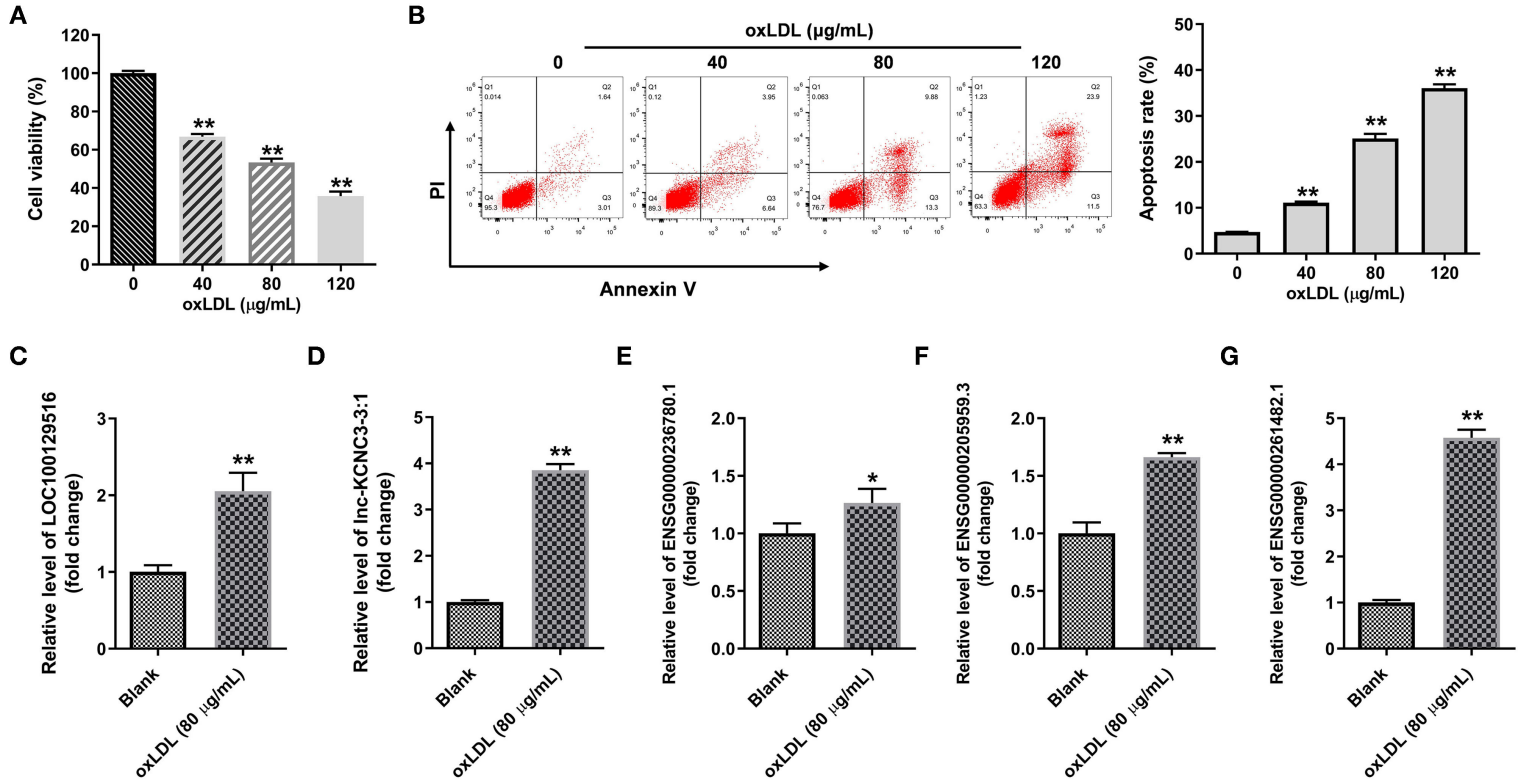
OxLDL increased the expression of LOC100129516, lnc-KCNC3-3:1 and ENSG00000261482. **(A)** HUVEC cells were treated with different concentrations (0, 40, 80, and 120 μg/mL) of oxLDL for 24 h. CCK-8 assay was used to detect the viability of HUVEC cells. **(B)** Flow cytometer assay was used to analyze HUVEC cells apoptosis. **(C–G)** RT-qPCR was used to detect the expression levels of LOC100129516, lnc-KCNC3-3:1, ENSG00000236780.1, ENSG00000205959.3 and ENSG00000261482.1. ^*^P < 0.05; ^**^P < 0.01 compared with control group, *n* = 3.

We next explore the role of top five differentially expressed lncRNA in the progression of atherosclerosis. To mimic atherosclerosis *in vitro*, HUVECs were treated with oxLDL (0, 40, 80, and 120 μg/mL) for 24 h and CCK-8 assay was used to detect the cell viability. As shown in Figures 2A,B, oxLDL dose dependently inhibited the proliferation of HUVEC by inducing apoptosis. Since 80 μg/mL of oxLDL exhibited about 50% inhibitory effect against cell growth; thus, 80 μg/mL oxLDL was utilized in the following experiments. In addition, RT-qPCR results indicated that oxLDL notably upregulated the expression of LOC100129516, lnc-KCNC3-3:1, ENSG00000236780.1, ENSG00000205959.3, or ENSG00000261482.1 in HUVEC cells. All these data suggested the top five differentially expressed lncRNA was upregulated as well in an *in vitro* model of atherosclerosis. Among these five lncRNAs, the increases of LOC100129516, lnc-KCNC3-3:1, and ENSG00000261482.1 were much higher in the *in vitro* model of atherosclerosis compared with the other two lncRNAs; thus, we focused on these 3 lncRNAs in the following experiments (Figures 2C–G).

### Knockdown of LOC100129516 or lnc-KCNC3-3:1 Significantly Reverses oxLDL-Induced Growth Inhibition of HUVEC

**Figure 3:**
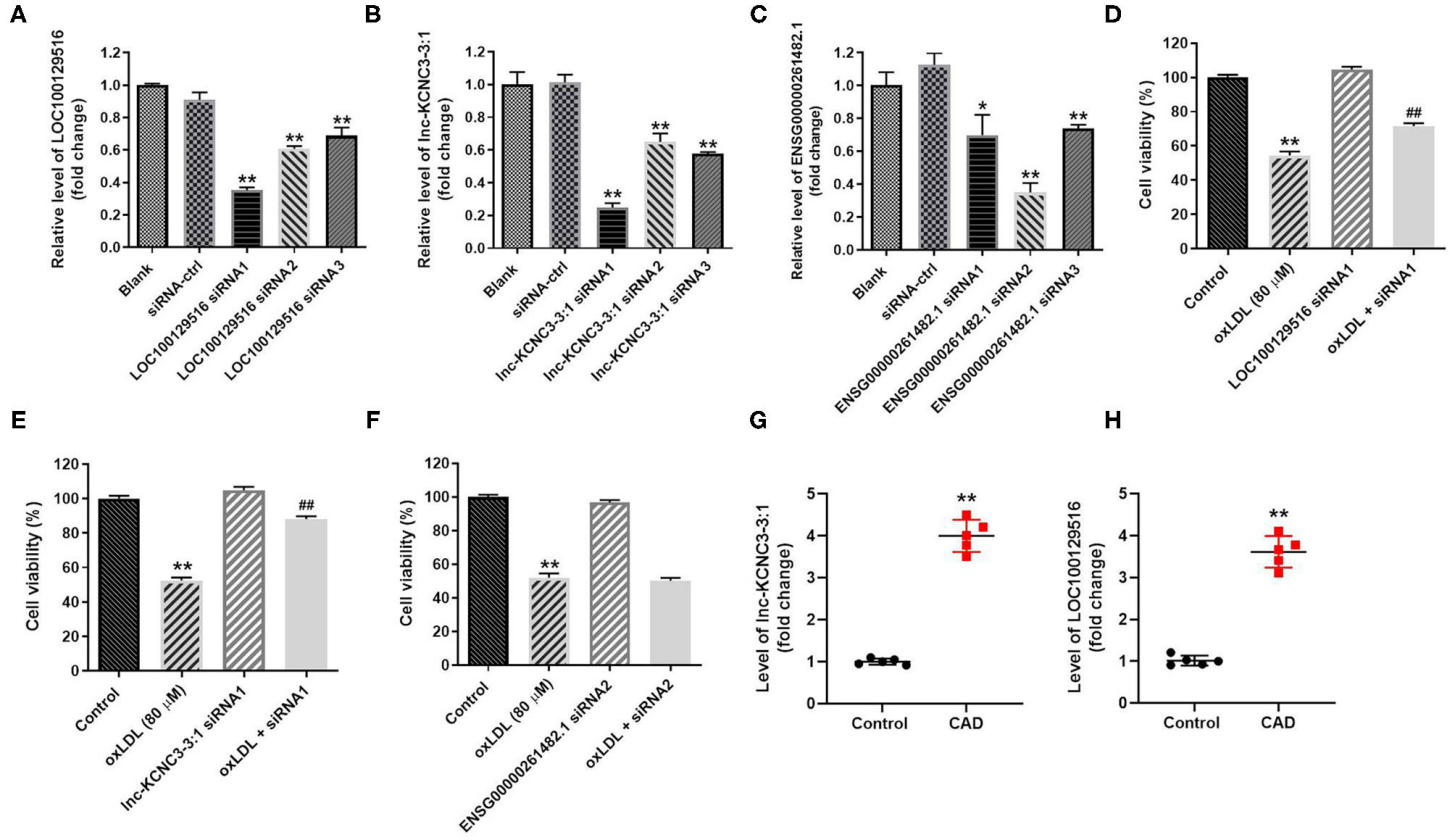
Knockdown of LOC100129516 or lnc-KCNC3-3:1 significantly reversed oxLDL-induced growth inhibition of HUVEC. **(A–C)** HUVEC cells were treated with 80 μg/mL of oxLDL for 24 h. The HUVEC cells were transfected with LOC100129516 siRNA1, LOC100129516 siRNA2, LOC100129516 siRNA3; lnc-KCNC3-3:1 siRNA1, lnc-KCNC3-3:1 siRNA2, lnc-KCNC3-3:1 siRNA3; ENSG00000261482.1 siRNA1, ENSG00000261482.1 siRNA2, ENSG00000261482.1 siRNA3 or siRNA-ctrl. The level of LOC100129516, lnc-KCNC3-3:1 or ENSG00000261482.1 in HUVEC cells was detected by RT-qPCR respectively. **(D–F)** The HUVEC cells were transfected with LOC100129516 siRNA1, lnc-KCNC3-3:1 siRNA1 or ENSG00000261482.1 siRNA1. CCK-8 assay was used to detect the viability of HUVEC cells. **(G,H)** The plasma samples from five patients with CAD and five healthy donators in the First Affiliated Hospital of Jinzhou Medical University were collected. The levels of lnc-KCNC3-3:1 and LOC100129516 in the plasma of a patient with CAD and healthy people was detected by RT-qPCR respectively. ^*^P < 0.05; ^**^P < 0.01 compared with control group. ^##^*P* < 0.01 compared with the oxLDL (80 μM) group, *n* = 3.

In order to explore the function of LOC100129516, lnc-KCNC3-3:1, and ENSG00000261482.1 in the progression of atherosclerosis, specific siRNAs were used. As revealed in RT-qPCR results, LOC100129516 siRNAs, lnc-KCNC3-3:1 siRNAs or ENSG00000261482.1 siRNAs successfully downregulated the level of LOC100129516, lnc-KCNC3-3:1 or ENSG00000261482.1 in HUVEC, respectively (Figures 3A–C). Since LOC100129516 siRNA1, lnc-KCNC3-3:1 siRNA1 or ENSG00000261482.1 siRNA2 exhibited the best inhibitory effect, which was selected of used in the following experiment (Figures 3A–C). In addition, the result of CCK-8 assay indicated knockdown of LOC100129516 or lnc-KCNC3-3:1 could reverse oxLDL-induced growth inhibition of HUVEC (Figures 3D–F); however, knockdown of ENSG00000261482.1 had no protective effect on cell growth. Moreover, RT-qPCR results showed that the levels of lnc-KCNC3-3:1 and LOC100129516 in the plasma of a patient with CAD were notably higher than that in the plasma of healthy people (Figures 3G,H). These results suggested that LOC100129516 and lnc-KCNC3-3:1 might play important roles during the occurrence and development of atherosclerosis.

### Knockdown of lnc-KCNC3-3:1 Reverses oxLDL-Induced Apoptosis of HUVEC

**Figure 4:**
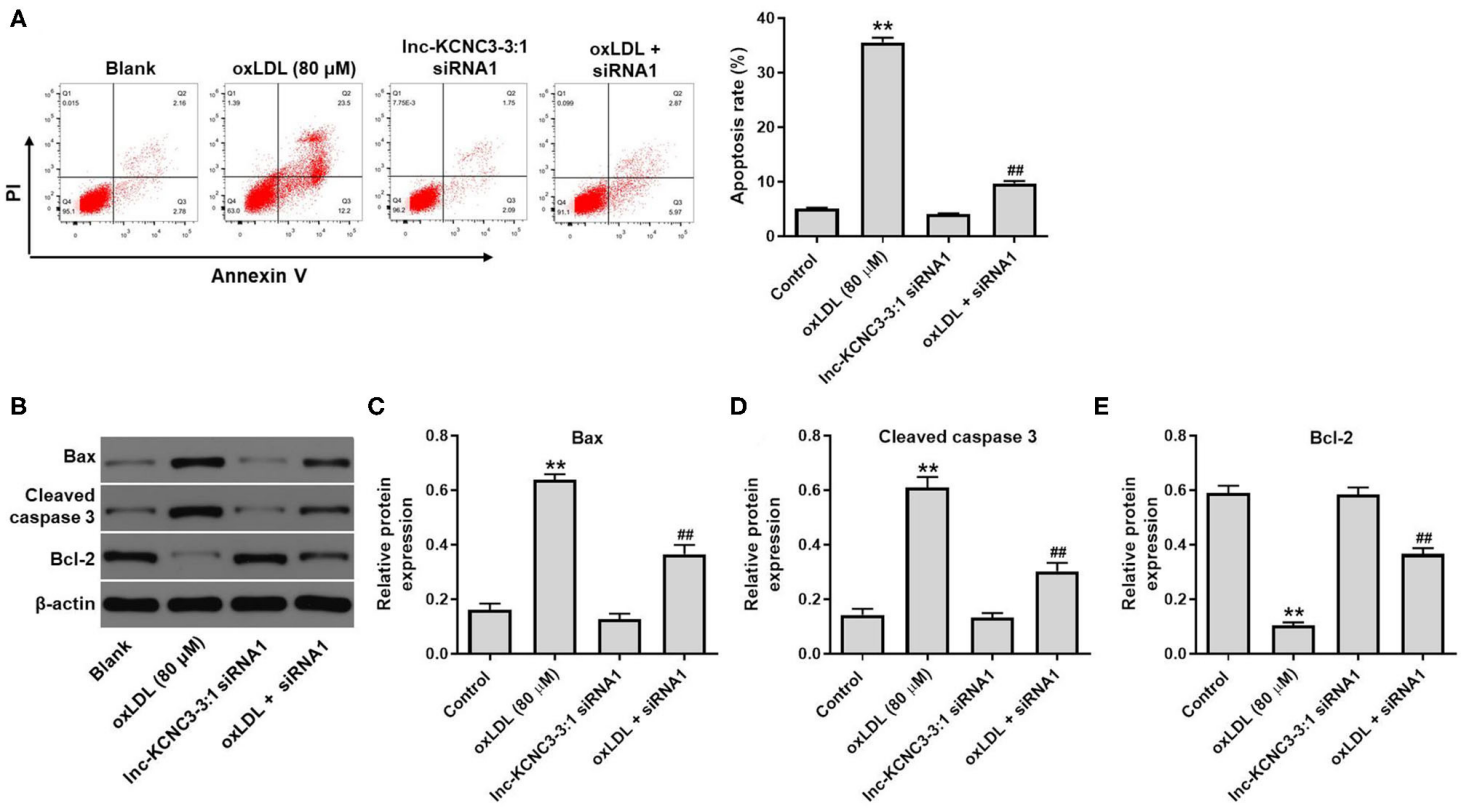
Knockdown of lnc-KCNC3-3:1 reversed oxLDL-induced apoptosis of HUVEC. HUVECs were transfected with lnc-KCNC3-3:1 siRNA1 for 6 h s. Then, cells were treated with 80 μg/mL of oxLDL for 24 h. **(A)** Flow cytometer assay was used to analyze the apoptosis of HUVEC. **(B–E)** Western blot was performed to measure the expressions of Bax, Bcl-2 and cleaved caspase 3 in HUVEC cells. ^**^P < 0.01 compared with control group. ^##^*P* < 0.01 compared with the oxLDL (80 μM) group, *n* = 3.

According to the result of the CCK-8 assay, lnc-KCNC3-3:1 knockdown exhibited much better protective effect against oxLDL compared with LOC100129516 knockdown (lnc-KCNC3-3:1 knockdown increased the viability of oxLDL-treated HUVECs from 50 to 88%. Meanwhile, LOC1000129516 knockdown increased the viability of oxLDL-treated HUVECs from 50 to 72%). Thus, we next focused on exploring the role of lnc-KCNC3-3:1 in the progression of atherosclerosis. The result of apoptosis assay indicated that oxLDL dramatically increased the apoptosis of HUVEC cells, while this phenomenon was notably reversed by lnc-KCNC3-3:1 siRNA1 (Figure 4A). As we know, Bax, Bcl-2 and cleaved caspase 3 are important proteins in the process of apoptosis (13, 14). Therefore, the levels of Bax, Bcl-2, and cleaved caspase 3 in cells were detected with Western blot. The outcome indicated that oxLDL obviously upregulated the levels of cleaved caspase 3 and Bax and downregulated the expression of Bcl-2; however, the effects of oxLDL on these proteins were all reversed by lnc-KCNC3-3:1 siRNA1 (Figures 4B–E). Taken together, knockdown of lnc-KCNC3-3:1 significantly reversed oxLDL-induced HUVEC apoptosis.

### Knockdown of lnc-KCNC3-3:1 Inhibits oxLDL-Induced Cell Migration *via* Downregulation of JAK1/STAT3 Pathway

**Figure 5:**
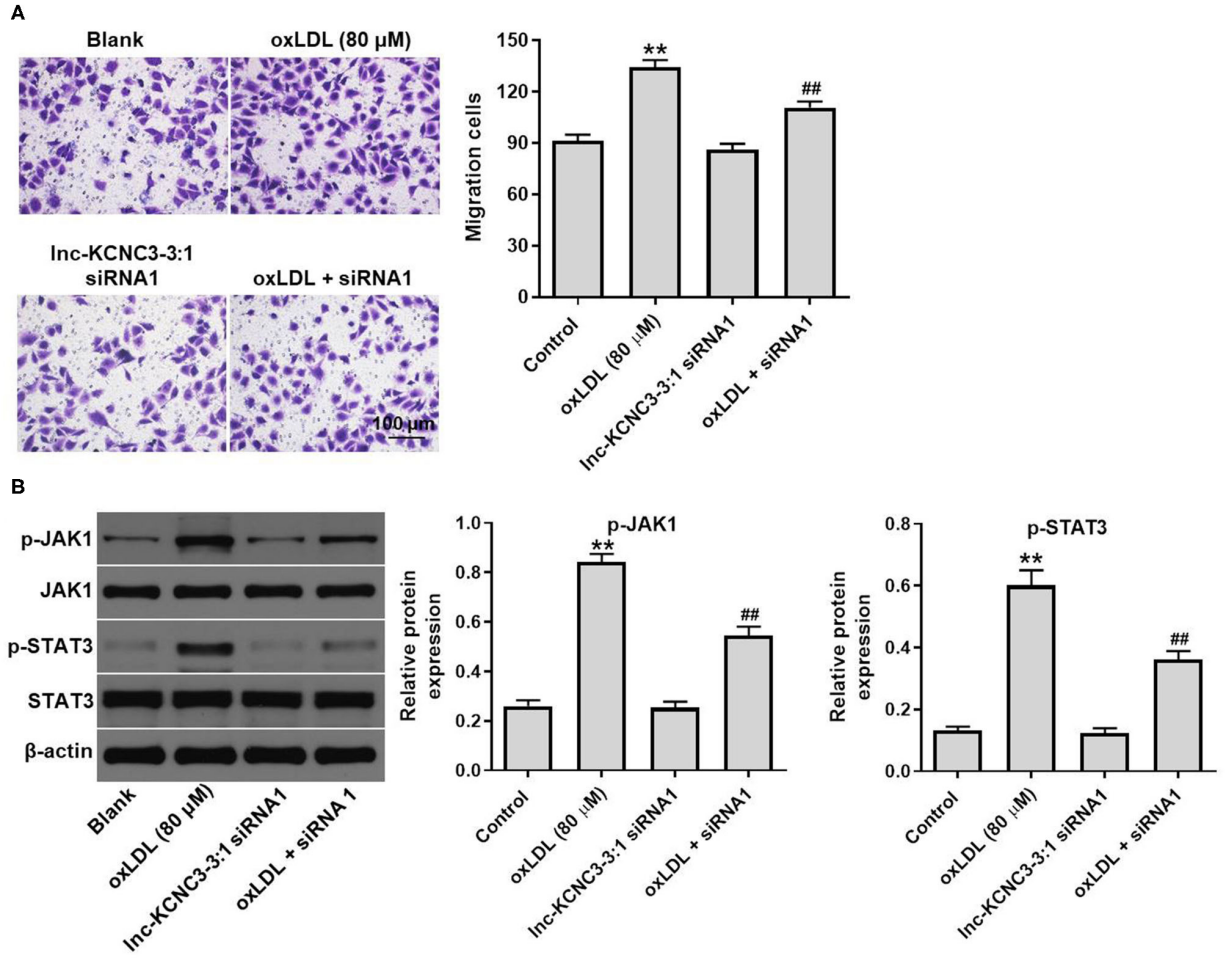
Knockdown of lnc-KCNC3-3:1 inhibits oxLDL-induced cell migration *via* downregulation of JAK1/STAT3 pathway. HUVECs were transfected with lnc-KCNC3-3:1 siRNA1 for 6 h s. Then, cells were treated with 80 μg/mL of oxLDL for 24 h. **(A)** Transwell migration assay was used to detect HUVEC cells migration ability. **(B)** Western blot was performed to measure the expressions of p-JAK1 and p-STAT3. ^**^P < 0.01 compared with control group. ^##^*P* < 0.01 compared with the atherosclerosis group, *n* = 3.

We next investigated the effect of lnc-KCNC3-3:1 knockdown on the migration of HUVEC. As indicated in Figure 5A, oxLDL dramatically promoted the migration ability of HUVEC, while this phenomenon was notably reversed by lnc-KCNC3-3:1 siRNA1. Moreover, lnc-KCNC3-3:1 might involve in JAK signaling pathways, according to the result of GSVA analysis (Figure 1D). Thus, we investigated the effect of lnc-KCNC3-3:1 siRNA1 on the expressions of p-JAK1 and p-STAT3 in HUVEC. Western blot result indicated that oxLDL notably increased the levels of p-JAK1 and p-STAT3 in HUVEC cells, whereas the increase of p-JAK1 and p-STAT3 was reversed in the presence of lnc-KCNC3-3:1 siRNA1 (Figure 5B). Taken together, these data suggest lnc-KCNC3-3:1 knockdown inhibited oxLDL-induced cell migration via downregulation of JAK1/STAT3 pathway.

### Knockdown of lnc-KCNC3-3:1 Alleviates the Symptom of Atherosclerosis *in vivo via* Downregulation of JAK1/STAT3 Signaling Pathway

**Figure 6:**
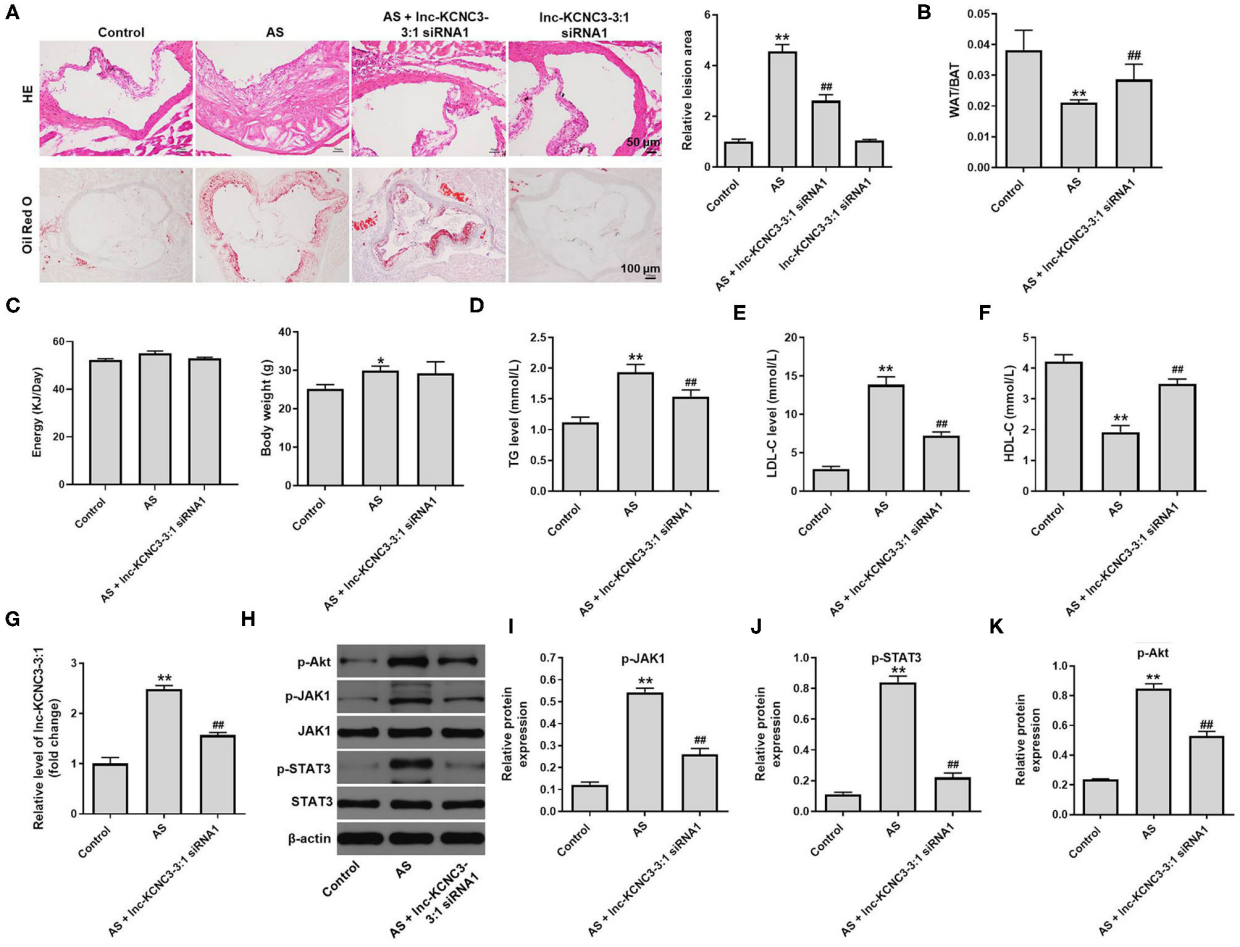
Knockdown of lnc-KCNC3-3:1 alleviates the symptom of atherosclerosis *in vivo* via downregulation of JAK1/STAT3 signaling pathway. **(A)** H&E and Oil-red O staining were used to observe the cross section of the aortic root. **(B)** The WAT/BAT in mice was calculated. **(C)** The body weight and appetite of mice were detected. **(D–F)** ELISA assay was used to measure the levels of TG, LDL-C and HDL-C in the plasma of mice. **(G)** RT-qPCR was performed to measure the level of lnc-KCNC3-3:1. **(H–K)** Western blot was performed to measure the expression of p-Akt, p-JAK1, JAK1, p-STAT3 and STAT3. ^**^P < 0.01 compared with control group. ^##^*P* < 0.01 compared with the atherosclerosis group, *n* = 6.

To further confirm the role of lnc-KCNC3-3:1 during the occurrence of atherosclerosis, *in vivo* atherosclerosis mouse model was established. H&E and Oil-red O staining were used to observe the cross section of the mouse arteries. In atherosclerosis model group, there was a significant increase in lipid accumulation and vascular wall thickening; however, these symptoms were notably alleviated by lnc-KCNC3-3:1 knockdown (Figure 6A). However, lnc-KCNC3-3:1 siRNA1 did not affect the lipid accumulation and vascular wall thickening (Figure 6A). Lnc-KCNC3-3:1 knockdown did not affect the body weight, appetite and WAT/BAT in AS mice (Figures 6B,C). It has been reported that an abnormally level of TG, LDL-C and HDL-C in the blood could be an underlying cause of atherosclerotic plaque (15–17). To investigate the level of TG, LDL-C and HDL-C in the plasma of mice, ELISA assay was used. The data indicated that the levels of TG and LDL-C were increased, and the level of HDL-C was decreased in atherosclerosis group; however, these changes were markedly reversed by lnc-KCNC3-3:1 siRNA1 (Figures 6D–F). In addition, RT-qPCR suggested that the expression of lnc-KCNC3-3:1 was notably upregulated in AS group, compared with the control group; however, this phenomenon was notably reversed by lnc-KCNC3-3:1 siRNA1 (Figure 6G). Consistent to *in vitro* data, the expressions of p-Akt, p-JAK1 and p-STAT3 in tissues of mice were notably increased in atherosclerosis group; however, these phenomena were completely reversed by lnc-KCNC3-3:1 siRNA1 (Figures 6H–K). All these results suggested that knockdown of lnc-KCNC3-3:1 alleviates development of atherosclerosis via downregulation of JAK1/STAT3 signaling pathway.

## Discussion

Atherosclerosis is a major cause of CAD, and CAD is one of the main causes leading to death in most countries (18). Evidences have shown that lncRNAs play critical roles in the development of atherosclerosis (1). The dataset GSE113079 from the GEO database showed that dysregulated lncRNAs in PBMCs of 93 patients with CAD and 48 healthy controls. Highly upregulated lncRNAs including LOC100129516, lnc-KCNC3-3:1 and ENSG00000261482.1 that were closely related to the occurrence and development of atherosclerosis were screened out.

In the current study, we found oxLDL obviously upregulated the levels of cleaved caspase 3 and Bax and downregulated the expression of Bcl-2; however, the effects of oxLDL on these proteins were all reversed by lnc-KCNC3-3:1 siRNA1. Bian et al. found that knockdown of lncRNA NORAD could promote the occurrence of atherosclerosis (19). In addition, Zheng et al. reported that silencing of lncRNA OIP5-AS1 could inhibit oxLDL-stimulated apoptosis in HUVEC cells by upregulating the level of Bcl-2 and downregulating the expressions of Bax and cleaved caspase 3 (20). Our finding is in line with these results.

It has been reported that the JAK/STAT signaling pathway play a important role in cell growth, activation, differentiation, apoptosis, inflammation, and pathogen resistance (18). In addition, it is well known that endothelial cell injury is the early event during the occurrence of atherosclerosis (1). In other words, atherosclerosis is a chronic inflammatory response of the arterial wall to endothelial cell damage (21, 22). For example, the expression of proteins associated with the JAK/STAT signaling pathway is notably upregulated in patients with atherosclerosis (23). Yang et al. found that the inhibition of JAK/STAT signaling pathway suppressed the progression of atherosclerosis (24). The results of current study suggested that lnc-KCNC3-3:1 siRNA1 markedly decreased the levels of p-JAK1 and p-STAT3 in vascular tissue of the atherosclerotic mouse. Moreover, Han et al. indicated that downregulation of lncRNA CRNDE leaded to the decreased expression of key proteins in the JAK/STAT signaling pathway (including JAK-1 and STAT-6), as well as the decreased expression of key angiogenesis related proteins (including VEGF and VEGFR2) in HUVEC cells (25). Our data are consistent with these results and suggested atherosclerosis is caused by endothelial cell damage due to the up-regulation of JAK/STAT signaling pathway. Collectively, knockdown of lnc-KCNC3-3:1 alleviates the symptom of atherosclerosis via downregulation of JAK1/STAT3 pathway. On the other hand, Akt phosphorylation could promote the cell growth (26). In our study, lnc-KCNC3-3:1 silencing could inhibit the phosphorylation of Akt in tissues of AS mice. In addition, previous studies have revealed that PI3K/AKT and JAK/STAT3 signaling pathways are involved in atherosclerosis (27, 28). Collectively, our data indicated that lnc-KCNC3-3:1 silencing inhibited the progression of atherosclerosis via inactivation of PI3K/Akt and JAK1/STAT3 signaling pathways.

Obliviously, there are some limitations in the current study. For example, the role of other differentially expressed lncRNAs in atherosclerosis progression did not unfold; other signaling pathways associated with atherosclerosis were not further investigated; downstream targets associated with the JAK1/STAT3 signaling pathway were not detected. The detailed mechanisms by which lnc-KCNC3-3:1 siRNA1 regulates lipid metabolism and the improvement of lipid profiles remain unclear. Therefore, more research is needed in the future.

Taken together, knockdown of lnc-KCNC3-3:1 alleviates the development of atherosclerosis via downregulation of JAK1/STAT3 signaling pathway. These data indicated that lnc-KCNC3-3:1 might serve as a potential target for the treatment of atherosclerosis.

## Data Availability Statement

The datasets presented in this study can be found in online repositories. The names of the repository/repositories and accession number(s) can be found in the article/supplementary material.

## Ethics Statement

This animal study was reviewed and approved by the Committee of the First Affiliated Hospital of Jinzhou Medical University. The studies involving human participants were reviewed and approved by Ethics Committee of the First Affiliated Hospital of Jinzhou Medical University. The patients/participants provided their written informed consent to participate in this study.

## Author Contributions

LS made major contributions to the conception, design, and manuscript drafting of this study. XH and TZ were responsible for data acquisition, data analysis, data interpretation, and manuscript revision. LS and GT made substantial contributions to conception and design of the study and revised the manuscript critically for important intellectual content. All authors agreed to be accountable for all aspects of the work, read, and approved the final manuscript.

## Conflict of Interest

The authors declare that the research was conducted in the absence of any commercial or financial relationships that could be construed as a potential conflict of interest.

## Publisher's Note

All claims expressed in this article are solely those of the authors and do not necessarily represent those of their affiliated organizations, or those of the publisher, the editors and the reviewers. Any product that may be evaluated in this article, or claim that may be made by its manufacturer, is not guaranteed or endorsed by the publisher.
